# Supplementary material for: The consequences of viral infection on protists
Source: Commun Biol. 2024 Mar 11;7:306. doi: 10.1038/s42003-024-06001-2 (PMC10925606; doi:10.1038/s42003-024-06001-2)
Supplement: Supplementary file 2 — Description of Add. Supp. files [file 42003_2024_6001_MOESM2_ESM.docx]

**Description of Additional Supplementary Files**

**File name:** Supplementary Data 1

**Description:** Table compiling all the information about the viruses and their respective hosts used to make figure 1.

**File name:** Supplementary Data 2

**Description:** Table compiling the list of publications mentioning cellular alterations derived from viral infections in protists used as references in the main text and as basis for making figure 2. Highlights of each paper are indicated.
